# Development and Assessment of Assisted Diagnosis Models Using Machine Learning for Identifying Elderly Patients With Malnutrition: Cohort Study

**DOI:** 10.2196/42435

**Published:** 2023-03-14

**Authors:** Xue Wang, Fengchun Yang, Mingwei Zhu, Hongyuan Cui, Junmin Wei, Jiao Li, Wei Chen

**Affiliations:** 1 Department of Clinical Nutrition Peking Union Medical College Hospital Chinese Academy of Medical Sciences and Peking Union Medical College Beijing China; 2 Institute of Medical Information Chinese Academy of Medical Sciences and Peking Union Medical College Beijing China; 3 Department of General Surgery Beijing Hospital National Center of Gerontology Beijing China; 4 Institute of Geriatric Medicine Chinese Academy of Medical Sciences Beijing China; 5 Department of Gastrointestinal Surgery Beijing Hospital National Center of Gerontology Beijing China

**Keywords:** disease-related malnutrition, global leadership initiative on malnutrition, GLIM, older inpatients, machine learning, Shapley additive explanation, SHAP, malnutrition, nutrition, older adult, elder, XGBoost, model, diagnose, diagnosis, diagnostic, visualization, risk, algorithm

## Abstract

**Background:**

Older patients are at an increased risk of malnutrition due to many factors related to poor clinical outcomes.

**Objective:**

This study aims to develop an assisted diagnosis model using machine learning (ML) for identifying older patients with malnutrition and providing the focus of individualized treatment.

**Methods:**

We reanalyzed a multicenter, observational cohort study including 2660 older patients. Baseline malnutrition was defined using the global leadership initiative on malnutrition (GLIM) criteria, and the study population was randomly divided into a derivation group (2128/2660, 80%) and a validation group (532/2660, 20%). We applied 5 ML algorithms and further explored the relationship between features and the risk of malnutrition by using the Shapley additive explanations visualization method.

**Results:**

The proposed ML models were capable to identify older patients with malnutrition. In the external validation cohort, the top 3 models by the area under the receiver operating characteristic curve were light gradient boosting machine (92.1%), extreme gradient boosting (91.9%), and the random forest model (91.5%). Additionally, the analysis of the importance of features revealed that BMI, weight loss, and calf circumference were the strongest predictors to affect GLIM. A BMI of below 21 kg/m2 was associated with a higher risk of GLIM in older people.

**Conclusions:**

We developed ML models for assisting diagnosis of malnutrition based on the GLIM criteria. The cutoff values of laboratory tests generated by Shapley additive explanations could provide references for the identification of malnutrition.

**Trial Registration:**

Chinese Clinical Trial Registry ChiCTR-EPC-14005253; https://www.chictr.org.cn/showproj.aspx?proj=9542

## Introduction

Epidemiological studies show that the proportion of aging people is expected to exceed 20% of the world's population by 2050, with 80% living in low- and middle-income countries [1]. As the second largest economy worldwide, China currently has the world's largest population with 1.44 billion people, which accounts for 19% of the global population, and China is swiftly changing into an aging country [2-4]. Older persons, usually defined by an age of 65 years or older, are at increased risk of malnutrition due to many factors. Malnutrition is related to poor outcomes, for example, increased rates of infections, length of hospital stay, duration of convalescence after acute illness, as well as mortality risk [5]. In the case of acute and chronic illness, nutritional problems are widespread, and a reduced dietary intake in combination with the effects of the catabolic disease rapidly leads to malnutrition [6]. According to the subject global assessment, the incidence of malnutrition among older hospitalized patients in China was higher than that of nonelderly hospitalized patients, at 32.98% and 22.19%, respectively [7].

Despite the high incidence and increased risk, a consensus diagnosis of malnutrition is not reached. One of the most important reasons is that the multidimensions need to be incorporated when assessing the nutritional status of inpatients. The global leadership initiative on malnutrition (GLIM) criteria advocate a “two-step” approach to diagnosing malnutrition, as follows: (1) screening the patients for nutrition risk by using validated nutrition screening tools, such as the nutritional risk screening 2002 (NRS-2002) and the Mini Nutritional Assessment–Short Form, and (2) for those who are at risk, diagnosing malnutrition if one of three phenotypic criteria (nonvolitional weight loss, low BMI, or reduced muscle mass) and one of two etiologic criteria (reduced food intake or assimilation and inflammation or disease burden) are met [8]. Research using large databases can be employed to achieve refinement of GLIM criteria. Machine learning (ML) is also introduced as a potential method to support the identification of the best cut points and combinations of operational criteria for use in the real world [9]. Previous studies proved the efficacy and clinical utility of these guidelines in identifying malnutrition in different diseases [10-13]. However, validation of the value of the GLIM guidelines in older patients remains insufficient [14,15]. There has not been a study that has evaluated the role of ML in validating the use of GLIM criteria in older patients.

Given the above background, this study was performed to explore the distribution of the core nutritional features in older patients to establish a decision-assisted system for assessing malnutrition and providing directions for individual intervention using ML approaches. Furthermore, we investigated the optimal cutoff value for important features, which could help aid the evaluation of nutritional diseases in the elderly.

## Methods

### Study Design and Population

This study was led by the Geriatric Nutrition Study Group of the Chinese Society for Parenteral and Enteral Nutrition, Chinese Medical Association. From June to September 2014, a clinical nutrition survey was conducted to assess the changes in nutritional status during hospitalization in 7122 Chinese patients from 34 large hospitals in 18 cities. The patients enrolled in this study were from a variety of clinical departments, including gastroenterology, respiratory medicine, neurology, oncology, general surgery, thoracic surgery, orthopedics, and geriatrics. The inclusion criteria were as follows: aged≥18 years; hospital stay of 7-30 days; no urgent surgery performed before 8 AM the day following admission; patients had to be conscious, willing to accept the multiple nutrition assessment, and have signed written informed consent. The patients belonging to the following categories were excluded from the examination: those who were younger than 18 years; with an expected hospital stay of <7 days or >30 days; unconscious; refused participation in the nutritional assessment or did not sign the written informed consent.

### Ethics Approval

**Figure 1:**
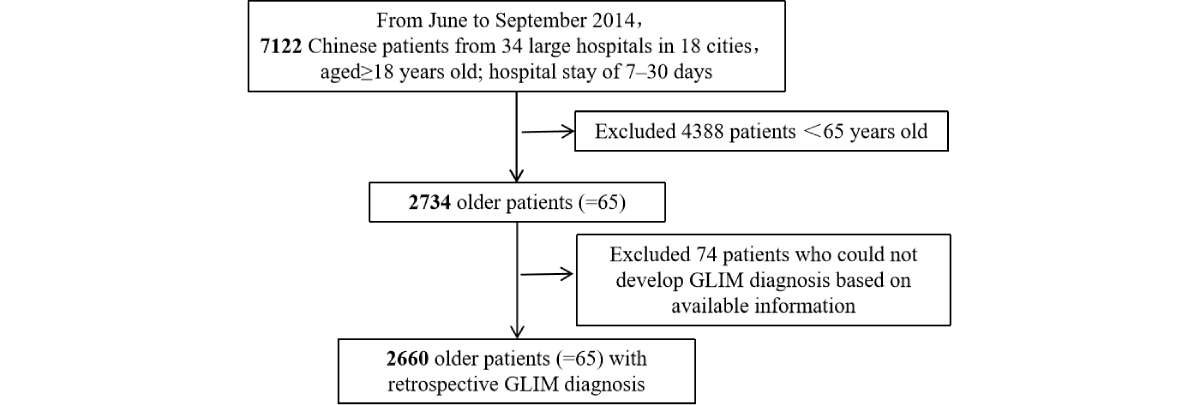
Flow diagram for the global leadership initiative on malnutrition (GLIM) cohort study.

The study protocol was approved by the Ethics Committee of Beijing Hospital (PIC approval number: 2014BJYYEC-022-02) and registered in the Chinese Clinical Trial Registry (No. ChiCTR-EPC-14005253). This study included only older adults (>65 years of age; n=2734 people). The flowchart of patients is shown in Figure 1.

### Diagnosis of Malnutrition Based on the GLIM Criteria

For participants at malnutrition risk (NRS2002≥3 in this study), at least one phenotypic criterion and one etiologic criterion should be met to establish the GLIM diagnosis: only if NRS2002 score is ≥3, GLIM score would be calculated. At least 1 phenotypic criterion and 1 etiologic criterion were assigned GLIM (+); the participants could be diagnosed with malnutrition; all phenotypic criteria and etiologic criteria were GLIM (-), and the diagnosis was negative. In addition to the above, the diagnosis can be “Not Known.” Participants who could not develop GLIM diagnosis were deleted. Please find the original scale of NRS2002 and GLIM in Multimedia Appendix 1.

In this study, the GLIM diagnosis was retrospectively determined according to the information collected in the study. A low BMI was diagnosed according to the Asian standards described in the GLIM criteria, and reference values for severe malnutrition were defined according to a previous study conducted among an Asian population [8,16]. Calf circumference was used to evaluate whether there was a reduced muscle mass. A value of less than 15% (percentile) according to a previous study (male<30 cm; female<29.5 cm) was defined as positive for moderate muscle loss [17]. Other criteria were derived from the results of NRS2002 (Table 1).

**Table 1:** Comparison of relevant criteria for nutritional risk screening 2002 (NRS2002) and global leadership initiative on malnutrition (GLIM)^a^.

Criteria			Items		Scores
**Phenotypic**					
	Weight change	No significant weight change or weight loss >5% in the last 6 months Weight loss >5% in the last 3 months Weight loss >5% in the last 2 months Weight loss >5% in the last 1 months		NRS2002=0， GLIM=Not known NRS2002=1， GLIM（+） NRS2002=2， GLIM（+） NRS2002=3， GLIM（+）	
	Low BMI	If ＜70 years, BMI＜18.5 kg/m2 If ＞70 years, BMI＜20 kg/m2		GLIM（+） GLIM（+）	
**Etiologic**					
	Food intake change	Normal Food-intake decrease of 25%-50% in 1 week Food-intake decrease of 50%-75% in 1 week Food-intake decrease of >75% in 1 week Food intake decrease in >2 weeks Any chronic gastrointestinal condition that adversely impacts food assimilation or absorption		NRS2002=0， GLIM（-） NRS2002=1， GLIM（-） NRS2002=2， GLIM（+） NRS2002=3， GLIM（+） GLIM（+） GLIM（+）	
	Disease burden or inflammation	Normal nutritional requirements Hip fractured, chronic cases, in particular with acute complications: cirrhosis, chronic hemodialysis, diabetes, and oncology Major abdominal surgery, stroke, severe pneumonia, and hematologic malignancy Head injury, bone marrow transplantation, and intensive care patients		NRS2002=0， GLIM（-） NRS2002=1，GLIM (+) NRS2002=2， GLIM（+） NRS2002=3， GLIM（+）	

^a^Only if NRS2002 score≥3, GLIM score would be calculated.

### Covariates and Outcome

In this study, feature selection was carried out in 2 steps. Firstly, based on recommendations and a review of literature, we chose 25 variables for the ML models, which included demographics (ie, gender, age, education, and type of Medicare), medical history (ie, weight loss, food consumption, gastrointestinal symptoms, and metabolic demand), physical examination (ie, subcutaneous fat, muscle consumption, edema, hand grip strength, BMI, and calf circumference), and laboratory test (ie, albumin, hemoglobin, white blood cells, lymphocyte count, total protein, total bilirubin, alanine transaminase, blood urea nitrogen, creatinine, platelet count, and glucose). According to the statistical differences between groups and expert opinions, 23 variables, except blood urea nitrogen (*P*=.65) and Creatinine (*P*=.66), were used to construct the prediction model [18,19].

### Data Processing

After checking for outliers and errors of data, we applied the imputation method for completing missing values using k-nearest neighbors. Each missing feature was imputed using values from the n nearest neighbors that have a value for the feature. The feature of the neighbors was averaged uniformly or weighted by distance to each neighbor. The development data set was split into training and test sets, with a ratio of 8:2. A stratified strategy was adopted for consistent outcome class proportions (normal nutrition vs abnormal nutrition) in the sub–data sets and data sets. Preprocessing was based on the features of the training set to avoid data leakage. Ordinal values for categorical variables were assigned to represent duration and converted into categorical values; categorical values were one-hot encoded. All other quantitative variables were standardized. After preprocessing, of 2660 patients diagnosed with malnutrition, 855 (32%) were divided as 684 (80%) into the training set, and 171 (20%) into the test set, respectively.

### Modeling and Interpretation

For training models, we applied 5 widely used ML algorithms in assisted diagnosis prediction , including logistic regression [20], tree-based model (light gradient boosting machine [LightGBM] [21]), random forest (RF) [22], and extreme gradient boosting (XGBoost) [23], multilayer perceptron [24], naive Bayes [25], and support vector machine–based methods [26]. The logistic regression model represents the linear model, which is a state-of-the-art classification model for a baseline construction. RF, LightGBM, and XGBoost were representative ensemble learning models, which do well in dealing with multiple types of features. RF is a bagging ensemble algorithm containing multiple decision trees, which uses the voting method to classify samples and integrate the final voting results produced by multiple decision trees. XGBoost is an optimized distributed gradient boosting library designed to be highly efficient, flexible, and portable. It implements ML algorithms under the gradient boosting framework. LightGBM is a gradient boosting framework that uses tree-based learning algorithms. It uses a gradient-based one-sided sampling algorithm to reduce the sample dimension and a mutually exclusive feature bundling algorithm to reduce the feature dimension.

After development, prediction performance was assessed using the area under the receiver operating characteristic curve (AUROC), sensitivity, specificity, and accuracy. To enhance the clinical applicability, we combined the diagnostic prediction results with clinical feature importance explanations to identify a patient with malnutrition. In this study, we used Shapley additive explanation (SHAP) values, to interpret feature contributions and assess the clinical significance of predictive models [27,28].

The SHA*P* value is the measurement of the marginal contribution of each feature in different combinations (Equation 1).




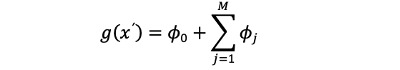




**Figure 2:**
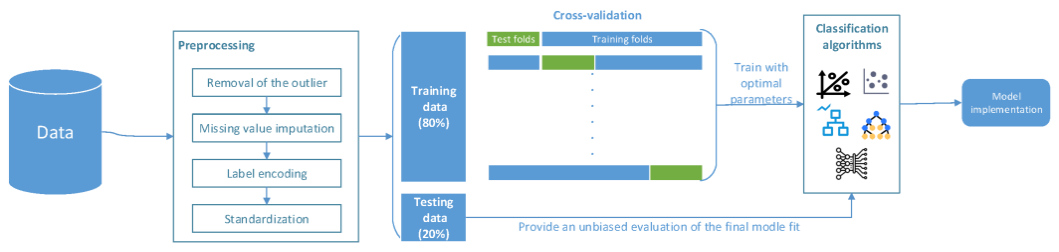
Workflow for the establishment and interpretation of malnutrition diagnosis tool using machine learning methods.

Where *ϕ_0_* is the average predicted value of all the samples, known as the base value, *ϕ_j_* is the SHA*P* value of the feature, and *M* is the total number of features. When *ϕ_j_* is greater than zero, this feature improves the predicted value and has a positive effect. According to the SHA*P* value, the effect direction and intensity of each feature can be obtained. Figure 2 shows the workflow for the establishment and interpretation of the malnutrition diagnosis tool.

For the ML model interpretation, we used both globally explainable and locally explainable methods [29]. The global explanation determines the importance of features by comparing the magnitude of the model prediction error change before and after replacing a feature. If the prediction error changes more, it indicates that the feature is more important. Meanwhile, we used SHA*P* values to explore individual-based decision-making processes in the view of local explanation.

Data preprocessing, model development, and external validation were performed in Python (version 3.8; Python Software Foundation). Model algorithms and hyperparameter tuning tools were based on Python scikit-learn (v0.24.2).

### Statistical Analysis

Continuous data were expressed as means (SD) and were compared using a *t* test. The normality of continuous data was tested using a Shapiro-Wilk test. Categorical data were expressed as n (%) and were compared using a chi-squared test. All tests were 2-tailed, and *P*<.05 was considered statistically significant. Statistical analyses were performed using SPSS Statistics (version 16; IBM Corp).

## Results

### Patient Characteristics

The GLIM criteria diagnosed 855 patients with malnutrition. GLIM diagnosed that malnutrition was significantly associated with age, BMI, hand grip strength, albumin, total protein, hemoglobin, lymphocyte count, total bilirubin, alanine transaminase, and platelet count (Table 2).

**Table 2:** Baseline characteristics of the study population (N=2660)

Characteristics		Overall	Normal (n=1805)	Malnutrition (n=855)	*P* value	
Age (years), mean (SD)		74.73 (7.11)	74.27 (7.19)	75.69 (6.85)	＜.001	
**Gender, n (%)**						.62
	Male	1584 (59.5)	1069 (59.2)	515 (60.2)		
	Female, n (%)	1076 (40.5)	736 (40.8)	340 (39.8)		
Type of Medicare, without insurance, n (%)		568 (21.4)	391 (21.7)	177 (20.7)	.57	
**Education, n (%)**						.52
	Primary school	1902 (71.5)	1286 (71.3)	616 (72.1)		
	Middle school	351 (13.2)	225 (12.5)	126 (14.7)		
	College	407 (15.3)	294 (16.3)	113 (13.2)		
BMI (kg/m^2^), mean (SD)		22.85 (3.60)	24.03 (3.09)	20.35 (3.31)	＜.001	
Hand grip strength (kg), mean (SD)		22.34 (9.48)	23.33 (9.51)	20.24 (9.06)	＜.001	
Calf circumference (cm), mean (SD)		32.52 (4.11)	33.53 (3.77)	30.41 (4.00)	＜.001	
Albumin (g/L), mean (SD)		38.18 (4.94)	39.13 (4.53)	36.17 (5.17)	＜.001	
Total protein (g/L), mean (SD)		65.50 (6.55)	66.22 (6.28)	63.96 (6.86)	＜.001	
Hemoglobin (g/L), mean (SD)		123.27 (20.11)	126.39 (18.55)	116.67 (21.66)	＜.001	
White blood cells (10^9^/L), mean (SD)		6.77 (3.03)	6.70 (2.74)	6.90 (3.56)	.13	
Lymphocyte count (10^9^/L), mean (SD)		3.67 (7.25)	4.13 (7.74)	2.72 (5.98)	＜.001	
Total bilirubin (μmol/L), mean (SD)		17.45 (34.98)	16.13 (29.52)	20.23 (44.24)	.005	
Alanine transaminase (U/L), mean (SD)		26.06 (43.37)	24.71 (38.12)	28.92 (52.67)	.02	
Platelet count (10^9^/L), mean (SD)		204.28 (78.64)	198.16 (70.59)	217.20 (92.11)	＜.001	
Glucose (mmol/L) mean (SD)		5.84 (1.98)	5.92 (1.98)	5.68 (1.96)	.005	
Weight loss within 6 months, n (%)		888 (33.4)	286 (15.8)	602 (70.4)	＜.001	
Reduced food intake, n (%)		1128 (42.4)	555 (30.8)	573 (67.0)	＜.001	
Gastrointestinal symptoms, n (%)		877 (33.0)	416 (23.1)	461 (53.9)	＜.001	
Increased metabolic demand, n (%)		1181 (44.4)	662 (36.7)	519 (60.7)	＜.001	
Edema, n (%)		186 (7.0)	91 (5.0)	95 (11.1)	＜.001	
Subcutaneous fat loss, n (%)		837 (31.5)	324 (18.0)	513 (60.0)	＜.001	
Muscle consumption, n (%)		786 (30.0)	302 (16.7)	484 (56.6)	＜.001	

### Evaluation of Model Performance

**Figure 3:**
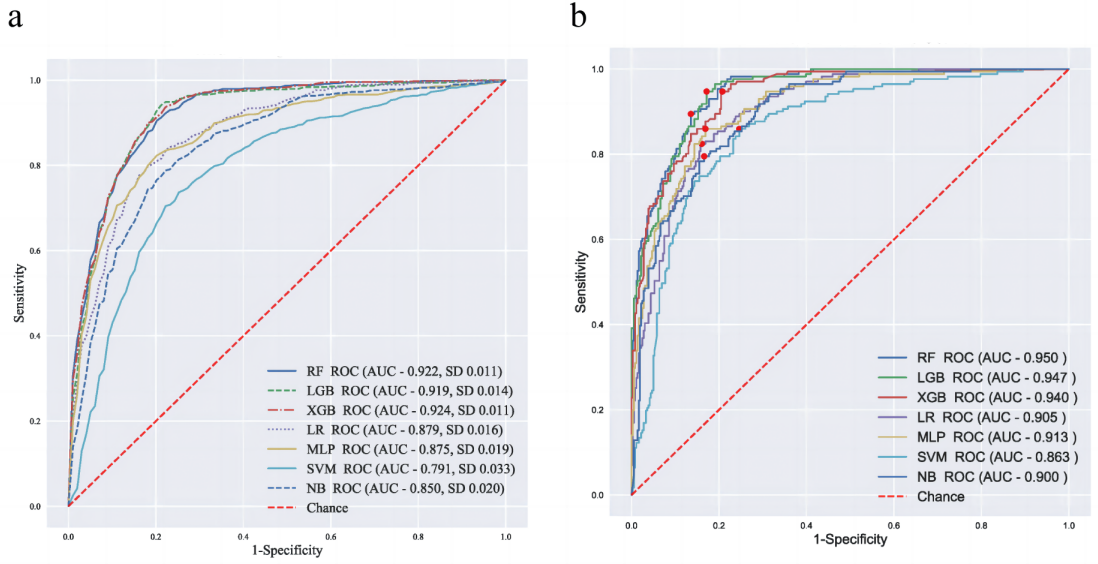
(a) Receiver operating characteristic curves of 10-fold cross-validation on the training set; (b) receiver operating characteristic curves in the test set. AUC: area under characteristic curve; LGB: light gradient boosting machine; LR: logistic regression; MLP: multilayer perceptron; NB: naive Bayes; RF: random forest; ROC: receiver operating curve; SVM: support vector machine; XGB: extreme gradient boosting.

The performance of predictive models was evaluated in terms of sensitivity, specificity, accuracy, and AUROC. In a 10-fold cross-validation performed on the training set, the XGBoost performed best in AUROC (92.5%), and logistic regression had the worst AUROC and second-worst sensitivity (Table 3). In the external validation cohort, the top 3 models by AUROC were the RF model (95.1%), LightGBM (94.7%), and XGBoost (94.0%), as shown in Table 4 and Figure 3.

**Table 3:** Performance metrics for prediction models of 10-fold cross validation on the training set.

	AUC^a^	Accuracy	Sensitivity	Specificity
RF^b^	0.923 (0.907-0.938)	0.743 (0.655-0.806)	0.633 (0.501-0.742)	0.974 (0.923-1.0)
LGB^c^	0.92 (0.893-0.941)	0.801 (0.764-0.817)	0.725 (0.669-0.754)	0.962 (0.942-0.982)
XGB^d^	0.925 (0.907-0.94)	0.798 (0.756-0.826)	0.721 (0.656-0.765)	0.961 (0.931-0.982)
LR^e^	0.879 (0.855-0.905)	0.756 (0.716-0.806)	0.696 (0.635-0.777)	0.882 (0.802-0.924)
MLP^f^	0.876 (0.846-0.901)	0.735 (0.639-0.795)	0.663 (0.51-0.775)	0.886 (0.821-0.953)
SVM^g^	0.791 (0.728-0.825)	0.631 (0.486-0.754)	0.561 (0.254-0.895)	0.778 (0.327-0.976)
NB^h^	0.851 (0.818-0.878)	0.787 (0.77-0.808)	0.796 (0.76-0.825)	0.769 (0.735-0.792)

^a^AUC: area under characteristic curve.

^b^RF: random forest.

^c^LGB: light gradient boosting machine.

^d^XGB: extreme gradient boosting.

^e^LR: logistic regression.

^f^MLP: multilayer perceptron.

^g^SVM: support vector machine.

^h^NB: naive Bayes.

**Table 4:** Performance metrics for prediction models in external test set.

	AUC^a^	Accuracy	Sensitivity	Specificity
RF^b^	0.950	0.701	0.560	1.00
LGB^c^	0.947	0.836	0.773	0.971
XGB^d^	0.940	0.831	0.773	0.953
LR^e^	0.905	0.767	0.693	0.924
MLP^f^	0.913	0.763	0.676	0.947
SVM^g^	0.863	0.724	0.634	0.912
NB^h^	0.900	0.812	0.814	0.807

^a^AUC: area under characteristic curve.

^b^RF: random forest.

^c^LGB: light gradient boosting machine.

^d^XGB: extreme gradient boosting.

^e^LR: logistic regression.

^f^MLP: multilayer perceptron.

^g^SVM: support vector machine.

^h^NB: naive Bayes.

### Identification of Important Risk Factors Contributing to the Model

To gain a thorough understanding of the general impact of features, the importance of features included in the XGBoost was quantified. Features and their subtypes were sorted by the magnitude of impact.

Categorical predictor variables were presented separately. The importance of features was sorted by the sum of the mean absolute value of the SHA*P* value magnitude over all samples. SHAP summary plots succinctly display the magnitude, prevalence, and direction of a feature's effect. As each dot corresponds to a single sample, the swarm plot avoids conflating the magnitude and prevalence of the feature effect into a single number. Therefore, apart from the magnitude of the features' global importance, it is informative concerning the exact impact on specific individuals.

**Figure 4:**
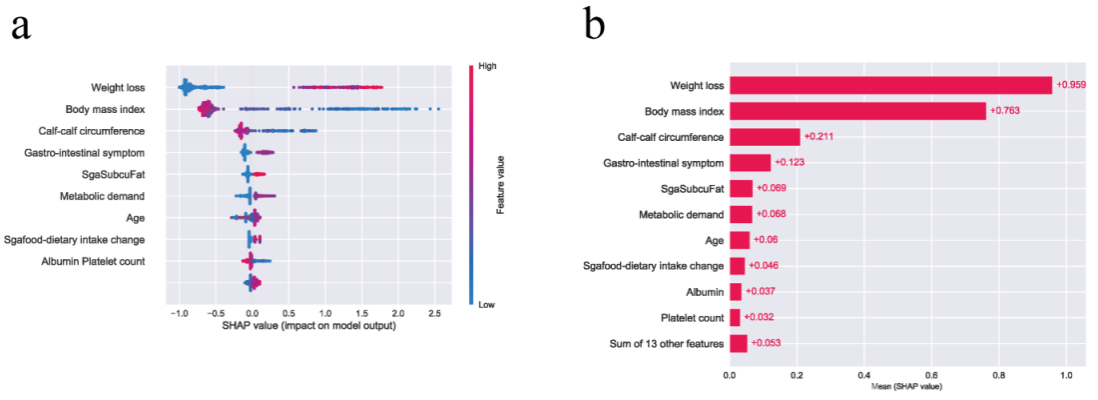
Model interpretation and visualization for (a) the relationship between features and global leadership initiative on malnutrition (GLIM) and (b) feature importance. SGA: small for gestational age; Sgafood: SGA-reduced food intake; SgaSubcuFat: SGA-subcutaneous fat; SHAP: Shapley additive explanations.

Figure 4(a) presents the feature importance as the strongest predictor to effect GLIM. Of the top 10 important features, there are 2 body measurement features, 4 laboratory features, and 4 nutrition-related histories. The results revealed that BMI, weight loss, and calf circumference were associated with a higher risk of GLIM events. Moreover, Platelet count and total protein also were important features. Additionally, we found that Lymphocyte count and Alanine transaminase seemed to be important features in predicting malnutrition.

Figure 4(b) summarizes the SHA*P* value plot by combining feature importance with feature effects. The y-axis is defined by the feature, and the x-axis is defined by the SHA*P* value. The plot describes the features’ overall influence on the model prediction. Each point in each feature represents an individual case, with colors ranging from blue (low feature value) to red (high feature value). The data points further to the right represent the features that contribute to the higher risk of malnutrition using GLIM for a given individual case. The data points to the left represent the features that contribute to the lower risk of GLIM. The vertical line in the middle represents no change in risk. We found that the data points (individual cases) with lower BMI values had a higher risk of GLIM.

**Figure 5:**
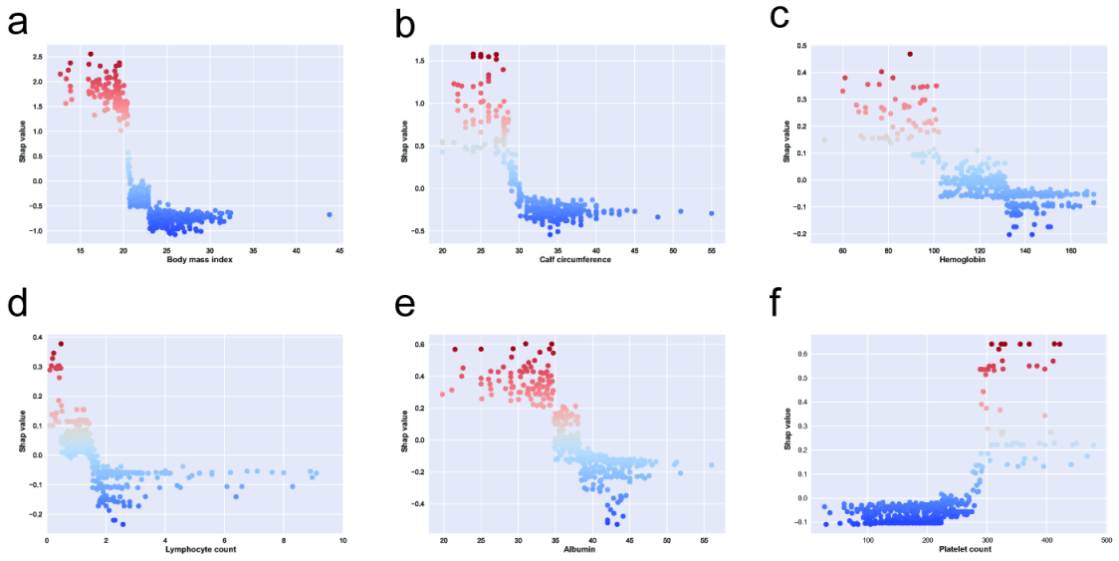
The Shapley additive explanations (SHAP) independence plot of body measurement features for (a) BMI, (b) calf circumference, and laboratory features for (c) hemoglobin, (d) lymphocyte count, (e) albumin, and (f) platelet count.

Furthermore, the lab tests and BMI were used as the continuous variable in our prediction model. This study examined the marginal effect of those features on the predicted outcome of an ML model using a SHAP dependence plot. As shown in Figure 5(a), the results showed that the value of BMI over approximately 21 kg/m^2^ was associated with a higher risk of GLIM. Patients had a high risk of GLIM when their platelet count value was below approximately 280×10^9^/L. As shown in Figure 5(b), when calf circumference was below 30, it increases the risk of GLIM. Moreover, hemoglobin below 100 increases the risk of GLIM.

**Figure 6:**
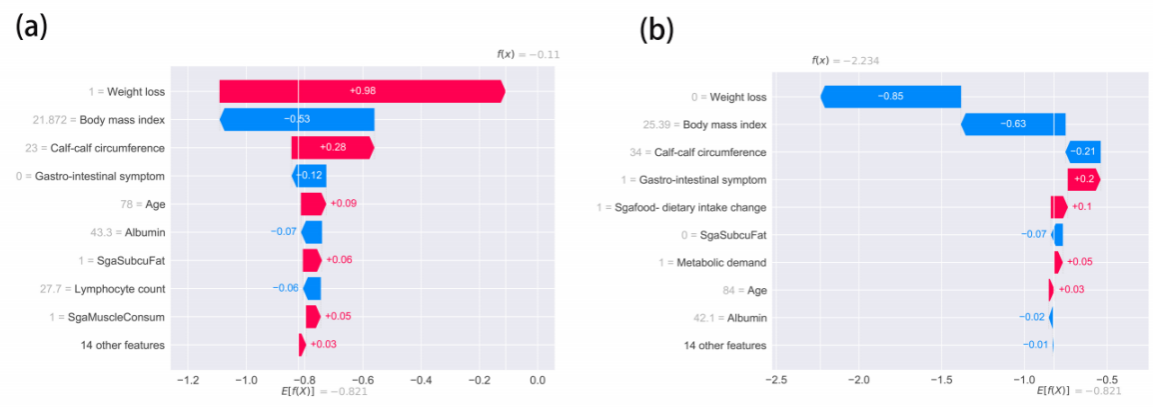
Local explanation for individual diagnostic interpretation: (a) features of patient with malnutrition and (b) features of an individual with normal nutrition. SGA: small for gestational age; SgaMuscleConsum: SGA-reduced muscle mass; SgaSubcuFat: SGA-reduced subcutaneous fat.

At the same time, we use the explainable method of ML to explain the constructed ML model. As shown in Figure 6, compared with previous studies, our method can not only propose diagnostic cutoff values for characteristic populations, but also interpret individual individuals, enabling medical workers to know in detail what each characteristic of a patient represents. As shown in Figure 6a, this is an individual diagnosed as malnourished by the machine model, and through the machine learning explainable method, we can clearly see that although his BMI is within the normal range, the characteristics of weight loss, calf circumference and age reflect that her nutritional status is not in a normal state, especially weight loss has a huge negative impact. At the same time, we can also provide health warnings for individuals identified as nutritionally healthy, as shown in Figure 6b, although the patient was diagnosed as nutritionally healthy by the machine learning model, her gastrointestinal symptoms and changes in eating habits have begun to increase her risk of malnutrition.

## Discussion

### Principal Findings

In this study, GLIM was retrospectively used to evaluate the nutritional status of older inpatients, and an assisted diagnosis model of malnutrition with a quite high performance was established by the ML algorithm. Using ML, we validated and visualized a tool that incorporated medical history and laboratory tests for identifying malnutrition in older inpatients based on the GLIM criteria. Through the SHA*P* value, we identified the most important features and their recommended cutoff values in evaluating malnutrition in older people. We also interpreted and visualized the model in individuals. Our model provided insights to the diagnosis of malnutrition and possesses the characteristic of high interpretability.

To the best of our knowledge, this is the first study using ML to validate nutritional status in older patients. The etiology of malnutrition in older individuals is multifactorial and consists of physiological, social, and economic parameters, often different from the young. Thus, it is important to develop a practical malnutrition tool and optimal cutoff values targeted to older people. The AUROC of the ML model achieved 0.921, which is comparable to findings by previous studies performed in different populations [17,18,30]. This could help clinicians acquire fast malnutrition diagnosis and implement interventions. According to the visualization of the model, the most important features are among phenotypic criteria in GLIM, that is, weight change, low BMI, and muscle mass. Furthermore, through the interpretation of individuals, we could also provide the extent to which individual risk factors influence diagnostic outcomes. This would allow doctors to know the focus of treatment more precisely. Compared with previous tools, our model could provide more individualized information to direct clinical intervention [17].

According to the analysis of the dependence plot, we could observe the cutoff values of the features with a higher risk of malnutrition. The cutoff point of BMI was approximately 21, which suggests that when BMI of older Chinese people is below 21 kg/m^2^, they are exposed to a higher risk for malnutrition. This coincides with the result from the current national BMI data set [19]. The results also provided helpful laboratory test information for identifying the risk of malnutrition. Based on the visualization for feature importance, albumin, platelet count, lymphocyte count, and hemoglobin were the top 4 laboratory tests.

### Limitations

This study has some limitations. First, the GLIM diagnosis was retrospectively determined. Although the original study collected a wide range of nutritional factors, more information was needed for accurate diagnosis and grading. In this study, GLIM-recommended muscle measurements such as dual-energy absorptiometry or bioelectrical impedance are not available; calf circumference was used as an alternative measure. In addition, we established the GLIM diagnosis based on Asia BMI thresholds, so the applicability of the model in a non-Asian population should be reevaluated. Furthermore, the training and test sets were derived from the same study, which means the model needs to be validated by prospective research. Lastly, the data were slightly imbalanced, which means the precision of the model training was relatively low because of the lower number of malnutrition cases.

### Conclusions

In conclusion, using ML, we could get an accurate and dynamic diagnosis of malnutrition based on the GLIM criteria. This model could be used as a decision tool to assist clinicians. The cutoff values of laboratory tests also provide references for the identification of malnutrition in Chinese older inpatients. Further studies are needed to simplify and validate the model in clinical conditions.

## Appendix

Multimedia Appendix 1 Supplementary figures.

## Abbreviations

AUROC: area under the receiver operating characteristic curve
GLIM: global leadership initiative on malnutrition
LightGBM: light gradient boosting machine
ML: machine learning
NRS-2002: nutritional risk screening 2002
RF: random forest
SHAP: Shapley additive explanations
XGBoost: extreme gradient boosting
