# Supplementary material for: Development and Assessment of Assisted Diagnosis Models Using Machine Learning for Identifying Elderly Patients With Malnutrition: Cohort Study
Source: J Med Internet Res. 2023 Mar 14;25:e42435. doi: 10.2196/42435 (PMC10131894; doi:10.2196/42435)
Supplement: Multimedia Appendix 1 [file jmir_v25i1e42435_app1.doc]

**Multimedia Appendix 1. Supplementary figures.**


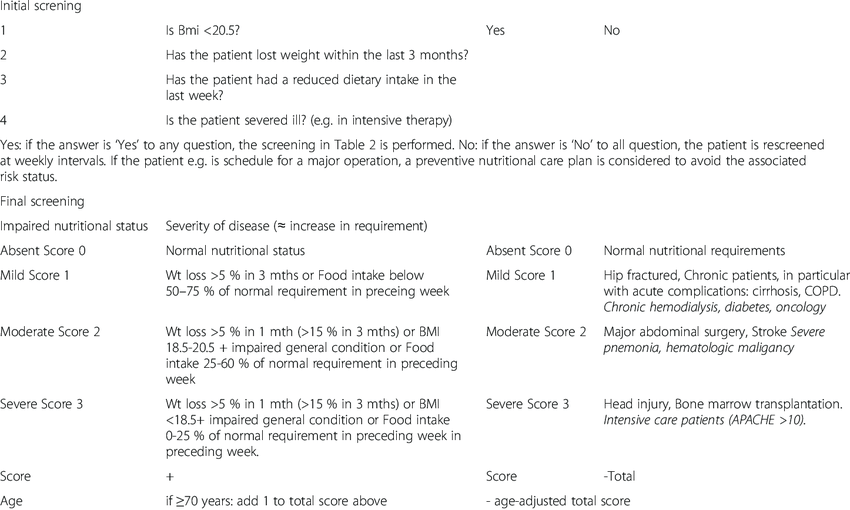


Figure S1. NRS-2002 scale


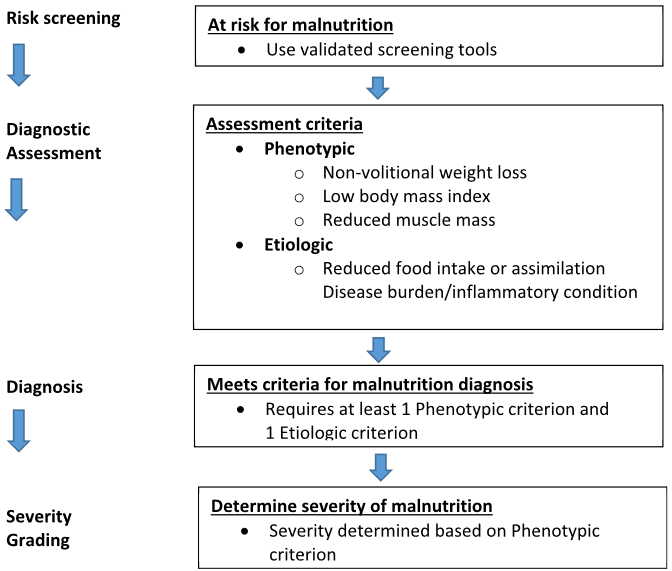


Figure S2. GLIM diagnostic scheme for screening, assessment, diagnosis and grading of malnutrition.
